# RISK OF FRAILTY ASSOCIATED WITH COGNITIVE DECLINE, SENSORY IMPAIRMENTS, AND FUNCTIONAL LIMITATION IN OLDER ADULTS

**DOI:** 10.1093/geroni/igac059.2124

**Published:** 2022-12-20

**Authors:** Jinhee Shin, Gwang Suk Kim, Agani Afaya

**Affiliations:** Yonsei University, Seoul, Seoul-t'ukpyolsi, Republic of Korea; Yonsei University, Seoul, Seoul-t'ukpyolsi, Republic of Korea; Yonsei University, Seoul, Seoul-t'ukpyolsi, Republic of Korea

## Abstract

The purpose of this study was to examine the effects and risk factors of cognitive decline, sensory impairment, and functional limitation associated with frailty among older adults. We conducted secondary data analysis using data from the Korean Longitudinal Study of Aging (2006–2018). The participants of this study consisted of 1,789 older adults over the age of 65 in South Korea who had never experienced frailty at the beginning of the survey (2006). Participants were classified into two groups, frailty and non-frailty, based on frailty events over time. We used Kaplan–Meier analysis to determine the effects of frailty-related group differences on cognitive decline (K-MMSE), sensory impairment (visual and hearing), and functional limitation (K-ADL) from 2006 to 2018. To determine the risk factors associated with frailty, the Cox regression analysis was performed. This study established that over time, approximately 71.2% of the older adults (n = 1,274) developed frailty. The study also revealed that over time, both physical (sensory impairment and functional limitation) and psychological factors (cognitive decline) could lead to frailty. We also identified that demographic (age, female), physical (BMI, chronic disease, hearing, vision, and K-ADL), and psychological (K-MMSE) factors were associated with frailty. This study provides an opportunity for healthcare professionals and policymakers to implement intervention programs tailored to ensure regular monitoring prevention and among of these risk factors.

